# Early Life Origins Cognitive Decline: Findings in Elderly Men in the Helsinki Birth Cohort Study

**DOI:** 10.1371/journal.pone.0054707

**Published:** 2013-01-30

**Authors:** Katri Raikkonen, Eero Kajantie, Anu-Katriina Pesonen, Kati Heinonen, Hanna Alastalo, Jukka T. Leskinen, Kai Nyman, Markus Henriksson, Jari Lahti, Marius Lahti, Riikka Pyhälä, Soile Tuovinen, Clive Osmond, David J. P. Barker, Johan G. Eriksson

**Affiliations:** 1 Institute of Behavioral Sciences, University of Helsinki, Helsinki, Finland; 2 Department of Chronic Disease Prevention, National Institute for Health and Welfare, Helsinki, Finland; 3 National Defence College, Finnish Defence Forces, Tuusula, Finland; 4 Centre for Military Medicine, Finnish Defense Forces, Lahti, Finland; 5 National Supervisory Authority for Welfare and Health, Helsinki, Finland; 6 MRC Epidemiology Resource Centre, University of Southampton, Southampton, United Kingdom; 7 Chair of Fetal Programming, King Saud University, Riad, Saudi Arabia; 8 Department of General Practice and Primary Health Care, Institute of Clinical Medicine, University of Helsinki, Helsinki, Finland; 9 Vasa Central Hospital, Vasa, Finland; 10 Unit of General Practice, Helsinki University Central Hospital, Helsinki, Finland; 11 Folkhälsan Research Centre, Helsinki, Finland; Cardiff University, United Kingdom

## Abstract

**Objectives:**

To examine whether the adverse effects of slow prenatal and postnatal growth on cognitive function persist to old age and predict age related cognitive decline.

**Design and Setting:**

A longitudinal birth cohort study of men born in Helsinki, Finland 1934-44.

**Participants:**

Nine-hundred-thirty-one men of the Helsinki Birth Cohort Study, with detailed data on growth from birth to adulthood, aged 20.1 (SD = 1.4) at the first and 67.9 (SD = 2.5) years at the second cognitive testing.

**Main Outcome Measures:**

The Finnish Defense Forces Basic Intellectual Ability Test assessed twice over nearly five decades apart.

**Results:**

Lower weight, length and head circumference at birth were associated with lower cognitive ability at 67.9 years (1.04–1.55 points lower ability per each standard deviation [SD] unit decrease in body size, 95% Confidence Interval [95%CI]: 0.05 to 2.72) and with cognitive decline after 20.1 years (0.07–0.11 SD decline over time per each SD decrease in body size, 95%CI:0.00 to 0.19). Men who were born larger were more likely to perform better in the cognitive ability test over time (1.22–1.43 increase in odds to remain in the top relative to the lower two thirds in ability over time per each SD increase in body size, 95%CI:1.04 to 1.79) and were more resilient to cognitive decline after 20.1 years (0.69 to 0.76 decrease in odds to decline from than remain in the top third of ability over time per each SD increase in body size, 95%CI:0.49 to 0.99). Slower growth between birth and two years in weight, height and body mass index was associated with lower cognitive ability at 67.9 years, but not with cognitive decline.

**Conclusions:**

Poorer lifetime cognitive ability is predicted by slower growth before and after birth. In predicting resilience to age related cognitive decline, the period before birth seems to be more critical.

## Introduction

Slow physical growth before and after birth predict poorer cognitive function in childhood, adolescence and young adulthood [1]–[13]. These associations are graded and extend across the whole range of body size. Findings from the British 1946 birth cohort suggest that early growth may not extend effects beyond young adulthood: lower birth weight predicted poorer cognitive function at ages eight, 11, 15 and 26 years, but it did not predict cognitive function at age 43 years [13].

However, studies focusing upon the long term effect of early growth on cognitive function across the whole life course are few. Findings from these studies are contradictory, and the studies have mostly focused on prenatal growth only [14]–[18]. Findings from these studies have shown that lower birth weight was associated with a lower test score on a test measuring concentration and calculation in 55 to 89-year-old women [14], and with a lower score on a test measuring verbal fluency in 51 to 70-year-old women and men [15]. Smaller bi-parietal head diameter at birth, but not birth weight, length, ponderal index, head circumference or occipitofrontal head diameter, was associated with a lower score on a test measuring fluid intelligence in 48 to 74-year-old women and men [16]. Birth weight and length were not associated with executive function, verbal and non-verbal reasoning, verbal declarative memory or general cognitive ability in 75 to 81-year-old women and men [17]. Neither were head circumference and ponderal index at birth associated with a lower general cognitive ability test score in 50 to 82-year old women and men [18], nor was head circumference at birth associated with logical memory or general cognitive ability in 66 to 75-year-old women and men [19]. Further studies on the long-term effects of physical growth before birth, but also those that focus on growth after birth are clearly warranted. The first objective of our study was to examine if body size, measured from birth to adult life, is associated with cognitive ability among men participating in the Helsinki Birth Cohort Study (HBCS) at the mean age of 67.9 years.

Cognitive ability test scores are shown to be highly stable from childhood to old age [20]. Therefore, it has been emphasized that in studies of cognitive aging it is important to take into account prior cognitive ability from an age where cognitive decline has not yet begun [17]. One previous study has shown that head circumference at birth was not associated with cognitive decline over 3.5 years in 66 to 75 year-old women and men [19], and two previous studies have shown that none of the physical prenatal growth parameters were associated with cognitive decline in 48 to 74-year-old [16] and 75 to 81-year-old women and men [17]. We are not aware of previous studies taking into account prior cognitive ability that would precede in time the stage of cognitive aging. Hence, our second study objective was to examine if body size, measured from birth to adult life, predicts age related decline in cognitive ability among men of the HBCS. We hypothesized that slower physical growth, both before and after birth, extends effects on poorer cognitive function up to old age and predicts a more rapid cognitive decline in adulthood.

## Methods

### Study population

The study cohort comprised men born at the Helsinki University Central Hospital during 1934-44. We identified 4630 men who had birth and child welfare clinic records and were still residents of Finland in 1971 when a unique personal identification number was allocated to each Finnish resident. Majority of them (77%) also went to school in Helsinki and had school health care records. The cohort has been described in detail elsewhere [21], [22].

Measurements of physical growth and cognitive ability at a mean age of 20.1 (standard deviation [SD] = 1.4; Range = 17.0–28.1) and 67.9 (SD = 2.5; Range = 64.5–75.7) years were available in 931 men. The mean time interval between the two cognitive tests was 47.7 (SD = 2.9, Range = 38.9–54.7) years. These men were identified from a subsample of 2786 (60% of the original cohort of men) men who performed their compulsory military service in the Finnish Defense Forces between 1952–1972, and who underwent an obligatory test on cognitive ability, usually within the first two weeks of their military service [11]. In year 2009, in late adulthood, 1750 men (62.8% of the subsample of 2786 men) were invited to a re-test and of them 931 participated (53.2% of the invited). Of the 1036 men who were not invited, 647 had died, 206 had in a previous follow-up study declined participation in any further follow-up, and 183 lived abroad or further than 200 km from Helsinki, in which case financial constraints prohibited their invitation, or we could not identify their current address.

The men who were invited to a re-test and who participated and the men who were not invited did not differ from each other in birth anthropometry, length of gestation, parity, history of breastfeeding or maternal characteristics (*P*-values>0.05). The invited men who participated weighed more, were taller from age two to 20.1 years, grew faster in height between age 11 and 20 years, more frequently had fathers with senior clerical occupations, more frequently had themselves attained an upper tertiary education in adulthood, were older at the first intellect testing, and earned a higher cognitive ability test score at first testing (*P*-values<0.03) when compared to the men who were not invited.

### Ethics Statement

Coordinating Ethics Committee of the Helsinki and Uusimaa Hospital District approved the study. The Finnish Defense Command gave permission for data linkage. All study participants signed a written informed consent.

### Anthropometric data

Weight (g), length (cm), body mass index (BMI; kg/m^2^) and head circumference (cm) at birth were extracted from hospital birth records. Monthly estimates between birth and two years and annual estimates between two and 11 years and at age 20.1 years of weight, height and BMI were derived from child welfare clinic, school and military records. Head circumference was not measured at these age stages. Weight, height, BMI and head circumference were measured again in conjunction with the cognitive ability re-test at 67.9 years.

### Cognitive function

The general cognitive ability test scores were obtained from the Finnish Defense Forces Basic Intellectual Ability Test. This test battery was administered to the participants twice over nearly five decades, during their compulsory military service at a mean age of 20.1 years and in a re-test at a mean age of 67.9 years.

This group-administered test battery measures verbal, arithmetic, and visuospatial reasoning and yields a general cognitive ability total score. Verbal, arithmetic and visuospatial reasoning subtests are timed and each subtest is composed of 40 multiple-choice questions that are ordered by difficulty. Verbal and arithmetic subtests comprise four types of questions. In the verbal reasoning test the subject has to choose synonyms or antonyms of a given word, a word belonging to the same category as a given word pair, which word of a word list does not belong in the group, and similar relationships between two word pairs. In the arithmetic reasoning test the subject has to complete a series of numbers that have been arranged to follow a certain rule, to solve verbally expressed short problems, to compute simple arithmetic operations, and to choose similar relationships between two pairs of numbers. The visuospatial reasoning subtest comprises a set of matrices containing a pattern problem with one removed part. Being analogous to Raven's Progressive Matrices [23], the subject is asked to decide which of the given single figures completes the matrix and requires the subject to conceptualize spatial relationships ranging from the very obvious to the very abstract. Correct answers to each subtest were summed, and their arithmetic mean was used as an index of general cognitive ability and logical thinking. The test battery and its psychometric properties have been described in detail elsewhere [11], [24].

### Covariates and confounders

In line with previous work from this [11], [25] and other cohorts [1]–[10], [12]–[18], adjustments were made for gestational age (from the date of the mother's last menstrual period), mother's age (years) and height (cm) at delivery and parity (primiparous vs. multiparous), social class in childhood based on father's occupational status (manual worker, lower middle class, upper middle class), history of breastfeeding (yes *vs.* no) extracted from birth, child wellbeing clinic or school records, highest own achieved level of education in adulthood (basic/primary or less, upper secondary, lower tertiary, upper tertiary) recorded at five-year intervals between 1970–2005 by Statistics Finland, and diagnoses of stroke (international classification of disease (ICD) codes 430–434 and 436–437 from ICD-8 and 9, 438 from ICD-9, and I60–I69 from ICD-10) [26] and coronary heart disease (CHD) (codes 410–414 from ICD-8 and ICD-9 and I21–I25 from ICD-10) [21] recorded between 1969–2008 in the Finnish Hospital Discharge Register.

### Statistical analysis

We first examined if body size at birth, and at two, seven, 11, 20.1 and 67.9 years, each age stage examined in a separate regression equation, were associated with cognitive ability at age 67.9 years. We then examined if growth from birth to two, seven, 11 and 20.1 years, where residual change scores modeling these growth periods were entered simultaneously into one regression equation, were associated with cognitive ability at age 67.9 years. Second, we examined if body size at birth, and at two, seven, 11, 20.1 and 67.9 years, and growth from birth to two, seven, 11 and 20.1 years were associated with age related decline in cognitive function after 20.1 years. As shown in Table 1 the sample size for these analyses varied by the number of men providing data on body size at each different measurement point and by the number of men providing data on cognitive ability at the two measurement points: data analyses were carried out in the maximal available sample size.

**Table 1:** Childhood and Adult Characteristics of Men Born in 1934–1944 and Followed-Up in 2009 in Helsinki, Finland.

Characteristic	N	Mean (SD)
**At Birth:**		
Weight–g	931	3482.4 (474.5)
Length–cm	920	50.7 (2.0)
Ponderal Index–kg/m^3^	919	26.6 (2.1)
Head circumference–cm	915	35.5 (1.5)
Gestational age–days	903	278.3 (12.7)
Mother's age at delivery–yr	930	28.5 (5.5)
Mother's height at delivery–cm	810	159.7 (5.7)
Parity (primiparous)–no. (%)	931	448 (48.0%)
Breastfeeding (yes)–no. (%)	931	773 (83.0%)
Father's occupational status in childhood	915	
Manual worker–no. (%)		494 (54.0%)
Lower middle class–no. (%)		221 (24.2%)
Upper middle class–no. (%)		200 (21.9%)
**At age 2 years:**		
Weight–kg	931	12.4 (1.1)
Height–cm	930	86.8 (3.1)
Body Mass Index–kg/m^2^	930	16.7 (1.2)
**At age 7 years:**		
Weight–kg	760	22.7 (2.5)
Height–cm	759	121.2 (4.7)
Body Mass Index–kg/m^2^	759	15.5 (1.1)
**At age 11 years:**		
Weight–kg	745	34.0 (4.3)
Height–cm	745	141.9 (5.6)
Body Mass Index–kg/m^2^	744	16.9 (1.4)
**At age 20.1 years:**		
Weight–kg	931	69.3 (8.9)
Height–cm	931	177.2 (5.8)
Body Mass Index–kg/m^2^	931	22.0 (2.4)
Cognitive ability (0–40 points) a,b	929	27.4 (6.6)
Bottom third (–25 points)	309	19.7 (4.4)
Middle third (–31 points)	303	28.4 (1.7)
Top third (>31 points)	317	34.0 (1.8)
**At age 67.9 years:**		
Weight–kg	931	85.5 (14.2)
Height–cm	931	175.0 (6.0)
Body Mass Index–kg/m^2^	931	27.9 (4.0)
Head circumference–cm	931	57.4 (1.5)
Cognitive ability (0–40 points) a	931	27.1 (6.1)
Bottom third (–25 points)	311	20.1 (4.2)
Middle third (–30 points)	308	28.2 (1.5)
Top third (>30 points)	312	33.2 (1.5)

Abbreviation: SD, standard deviation.

a Cognitive ability raw test score is the arithmetic mean of three subtests measuring verbal, arithmetic and visuospatial reasoning.

b For two men the cognitive ability test score was set as missing, as they did not complete one of the three cognitive ability subtests.

Measurements of body size at each age stage were converted into z scores with a mean of 0 and SD of 1, and cognitive ability test score at 67.9 years was converted into a z score with a mean of 100 and SD of 15. Postnatal growth variables were standardized residual change scores from linear regression models where weight, height and BMI at two, seven, 11 and 20.1 years were regressed on corresponding measures at all earlier time points creating completely uncorrelated residuals reflecting growth conditional on previous history [26]. A standardized residual change score, derived from a linear regression analysis where cognitive ability at 67.9 years was regressed on cognitive ability at 20.1 years, was used as the outcome in the analyses of age related cognitive decline. The standardized residual change scores have a mean of 0 and SD of 1. The standardized scores represent the difference from the mean value for the entire sample of men participating in the current study.

**Figure 1:**
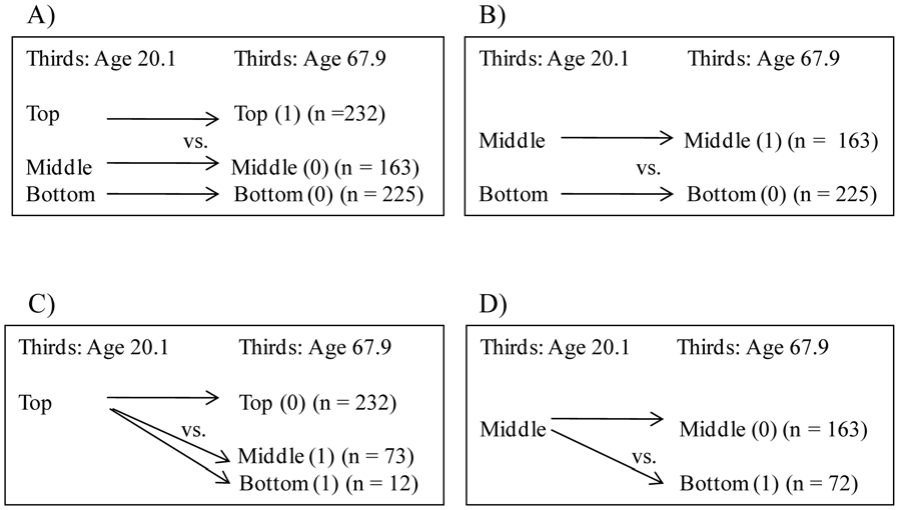
Schematic illustration of logistic regression analyses of cognitive development after age 20.1 years. Schematic illustration of logistic regression analyses comparing men who remained in the top, middle and bottom thirds (panels A and B) and who declined from the top and middle thirds in cognitive ability after age 20.1 years (panels C and D).

We also used logistic regression analyses to delineate further the associations of body size at birth, and at two, seven, 11, 20.1 and 67.9 years, and growth from birth to two, seven, 11 and 20.1 years with cognitive ability from 20.1 to 67.9 years (Figure 1). In these analyses cognitive ability test scores at ages 20.1 and 67.9 years were first categorized into tertiles. Then, body size at each age stage and growth from birth to 20.1 years of men who remained in the top third of cognitive ability over time were compared (a) with men who remained in the lower two thirds of cognitive ability (Figure 1, panel A), and (b) with men whose cognitive ability test scores declined from the top third to the lower two thirds (Figure 1, panel C). Finally, body size at each age stage and growth from birth to 20.1 years of men who remained in the middle third of cognitive ability over time were compared (c) with men whose cognitive ability test scores remained in the bottom third (Figure 1, panel B), and (d) with men whose cognitive ability test scores declined from the middle third to the bottom third (Figure 1, panel D). This analysis allowed us to compare men who had an equally high baseline level of cognitive ability and who over time up to old age either remained in this equally high category or declined from this category.

We made adjustments for length of gestation (birth measures), father's occupational status in childhood, mother's age and height at delivery, parity, history of breastfeeding, age at cognitive ability test, and time interval between the two cognitive ability tests (analyses of change). Further models adjusted for highest own achieved level of education in adulthood and for diagnoses of stroke and CHD (n = 108; 31 had had stroke, 69 had had CHD only, 8 had had both). All data were analyzed using PASW Statistics 18 package.

## Results

Table 1 shows characteristics of the sample. The unadjusted intra-class correlation between the two cognitive ability test scores measured across 47.7 years was 0.87 (*P*<0.001). After adjusting for age at first cognitive ability test and time interval between the two tests, cognitive ability showed high rank-order stability (standardized regression coefficient = 0.78, *P*<0.001); the average age-related mean-level change was −0.23 (SD = 4.57, Range = −0.33–−18.0, *P* = 0.11) raw test score points; 52.3% of the sample displayed a decline in cognitive ability after 20.1 years.

Of the covariates and confounders, lower cognitive ability at age 67.9 years was associated to father's lower occupational status in childhood, multiparity, not being breastfed, older age at testing, lower own maximum achieved level of education in adulthood, and diagnoses of stroke or CHD (*P*-values<0.03); Cognitive ability at 67.9 years was not associated with length of gestation or mother's age and height at delivery (*P*-values>0.43). Decline in cognitive ability after 20.1 years was associated with older age and longer time interval between the two cognitive tests, lower own maximum achieved level of education in adulthood, and diagnoses of stroke or CHD (*P*-values<0.01), but it was not associated with the other covariates and confounders (*P*-values>0.36).

Table 2 presents the analyses testing if body size at birth and at ages two, seven, 11 and 20.1 years, body size at each age stage examined in a separate regression equation, associated with cognitive ability at age 67.9 years. Each one SD unit (1 SD) lower weight, length and head circumference at birth was associated, respectively, with 1.04, 0.96 and 0.97 points lower cognitive ability (Table 2, adjusted *P*-values < 0.05). Also, lower weight and/or BMI at two and seven years, shorter height at 20.1 and 67.9 years, and smaller head circumference at 67.9 years were associated with lower cognitive ability (Table 2, adjusted *P*-values<0.05).

**Table 2:** Body size at birth, and in child-and adulthood and cognitive ability at 67.9 years.

	Cognitive ability in standardized points a					
Body size (unconditional on previous history) in standard deviation units at (years):	Unadjusted			Adjusted b, c, d		
	Regression coefficient e	95% CI	P	Regression coefficient e	95% CI	P
Weight						
Birth	1.04	0.05, 2.03	0.04	1.31	0.06, 2.55	0.04
2	2.39	1.39, 3.38	<0.001	1.93	0.85, 3.01	<0.001
7	2.36	1.21, 3.50	<0.001	1.61	0.35, 2.88	0.01
11	2.08	0.91, 3.25	0.001	0.95	−0.35, 2.24	0.15
20.1	1.92	0.92, 2.90	<0.001	1.19	0.10, 2.28	0.03 c
67.9	−0.38	−1.35, 0.58	0.44	−0.57	−1.58, 0.44	0.27
Length/Height						
Birth	0.96	0.01, 1.91	0.05	1.43	0.27, 2.58	0.02
2	1.58	0.59−2.56	0.002	1.09	−0.01, 2.18	0.05 c
7	1.84	0.73−2.95	0.001	0.97	−0.31, 2.25	0.14
11	1.84	0.68−3.00	0.002	1.01	−0.30, 2.32	0.13
20.1	1.87	0.86−2.89	<0.001	1.61	0.41, 2.80	0.01
67.9	2.09	1.13−3.04	<0.001	1.81	0.70, 2.93	0.001
Ponderal index/Body mass index						
Birth	0.53	−0.48, 1.54	0.31	0.19	−0.93, 1.30	0.74
2	1.57	0.58, 2.55	0.002	1.41	0.39, 2.42	0.01
7	1.46	0.36, 2.56	0.01	1.30	0.15, 2.46	0.03
11	1.22	0.08, 2.37	0.04	0.25	−0.95, 1.45	0.68
20.1	1.16	0.15, 2.17	0.02	0.54	−0.53, 1.61	0.32
67.9	−1.31	−2.27, −0.35	0.01	−1.19	−2.18, −0.20	0.02 c
Head circumference f						
Birth	0.97	−0.02, 1.97	0.05	1.55	0.39, 2.72	0.01
67.9	1.22	0.26, 2.18	0.01	1.09	0.07, 2.10	0.04

Abbreviations: CI, confidence interval;

a Cognitive ability test score was standardized and has a mean of 100 and a standard deviation of 15.

b The adjusted model refers to adjustments made for length of gestation (birth measures), father's occupational status in childhood, parity, mother's age and height at delivery, history of breastfeeding, and age at testing cognitive ability at 67.9 years.

c A further model adjusted for ‘*^b^*’ plus highest own achieved level of education. The marked associations were rendered non-significant. Unmarked associations remained as in the adjusted model ‘*^b^*’.

d A further model adjusted for ‘*^b^*’ plus diagnoses of stroke and coronary heart disease. The marked associations were rendered non-significant. Unmarked associations remained as in the adjusted model ‘*^b^*’.

e Regression coefficients, derived from linear regression analyses, reflect lower cognitive ability in standardized points (a) per each one standard deviation unit lower body size if the coefficient is positive, and (b) per each one standard deviation unit higher body size if the coefficient is negative.

f Measurements of head circumference at two, seven, 11 and 20 years were not available.

Analyses testing if postnatal growth from birth to two, seven, 11 and 20.1 years, with all standardized residual change scores of growth entered simultaneously into one regression equation, were associated with cognitive ability at 67.9 years, showed that each 1 SD slower gain in weight, height and BMI between birth and two years was associated, respectively, with 2.03 (95% Confidence interval [CI]: 0.94 to 3.13, *P*<0.001 in unadjusted model; adjusted *P*-values<0.03), 1.17 (95% CI: 0.06 to 2.29, *P* = 0.04; adjusted *P*-values>0.23) and 1.35 points (95% CI: 0.25 to 2.44, *P* = 0.02; adjusted *P*-values<0.04) lower cognitive ability at 67.9 years.

Table 3 shows how body size at birth was associated with age related decline in cognitive ability after age 20.1 years. Per each 1 SD lower weight, length and head circumference at birth, the decline in cognitive ability was, respectively, 0.07, 0.07 and 0.06 SD units. After adjustments, the association of birth weight was rendered to a moderately significant trend (adjusted *P*-values<0.06), the association of birth length remained unaltered, and the association of head circumference became significant from a non-significant trend (adjusted *P*-values<0.05) (Table 3).

**Table 3:** Body Size at Birth and Age Related Decline in Cognitive Ability After Age 20.1 Years.

Body size at birth in standard deviation units:	Decline in cognitive ability over five decades^a^					
	Unadjusted			Adjusted b, c, d		
	Regression coefficient	95% CI	P	Regression coefficient	95% CI	P
Weight	0.07	0.00, 0.14	0.04	0.08	−0.00, 0.17	0.06
Height	0.07	0.01, 0.14	0.03	0.10	0.02, 0.18	0.02
Ponderal Index	0.04	−0.03, 0.11	0.27	0.02	−0.06, 0.10	0.59
Head circumference	0.06	−0.01, 0.13	0.07	0.11	0.03, 0.19	0.01

Abbreviations: CI, confidence interval.

a Decline is a standardized residual score from a linear regression analysis where cognitive ability at age 67.9 years is predicted by cognitive ability at age 20.1 years.

b Adjustments were made for length of gestation, father's occupational status in childhood, parity, mother's age and height at delivery, history of breastfeeding, age at testing cognitive ability at 20.1 years, and time interval between tests of cognitive ability from 20.1 to 67.9 years.

c A further model adjusted for ‘b’ plus highest own achieved level of education. Unmarked associations remained as in the adjusted model ‘b’.

d A further model adjusted for ‘b’ plus diagnoses of stroke and coronary heart disease. Unmarked associations remained as in the adjusted model ‘b’.

Analyses testing if body size at two, seven, 11, 20.1 and 67.9 years, or growth from birth to 20.1 years were associated with age related cognitive decline revealed no significant associations (*P*-values>0.08). Except, men whose scores declined more after 20.1 years were shorter at 67.9 years (per each 1 SD shorter height cognitive ability declined by 0.09 SD units, 95% CI: 0.05 to 0.16, *P* = 0.005, adjusted *P*-values<0.04).

Table 4 presents the results from the logistic regression analyses comparing body size at birth and at two, seven, 11, 20.1 and 67.9 years of men whose cognitive ability test scores remained in the top third over time with men whose cognitive ability test scores remained in the lower two thirds over time (Figure 1, panel A). Higher weight and larger head circumference at birth were associated with higher odds for remaining in the top relative to the lower two thirds in cognitive ability after age 20.1 years (Table 4, adjusted *P*-values<0.05). The odds for remaining in the top third after age 20.1 years was also higher for men who weighed more at two, seven, 11 and 20.1 years, were taller at each age stage to 67.9 years, and had a larger head circumference at 67.9 years (Table 4; adjusted *P*-values<0.05). Further, the odds for remaining in the top third after age 20.1 years was higher for those men who gained weight (OR = 1.51, 95% CI: 1.23 to 1.85, *P*<0.001 per each 1 SD unit faster growth; adjusted *P*-values<0.01), height (OR = 1.34, 95% CI: 1.10 to 1.63, *P* = 0.004; adjusted *P*-values < 0.02) and BMI (OR = 1.26, 95% CI: 1.04 to 1.54, *P* = 0.02, adjusted *P*-values<0.05) faster between birth and two years.

**Table 4:** Body Size at Birth and in Child-and Adulthood and Odds for Retaining Top Scores in Cognitive Ability Over Five Decade^a^.

Body size (unconditional on previous history) in standard deviation units at (years):	Cognitive ability test scores in: Top third *versus* middle and bottom thirds at age 20.1 and 67.9 years					
	Unadjusted			Adjusted b,c,d		
	OR	95% CI	P	OR	95% CI	P
Weight						
Birth	1.22	1.04, 1.44	0.02	1.36	1.07, 1.74	0.01
2	1.59	1.33, 1.91	<0.001	1.65	1.32, 2.06	<0.001
7	1.60	1.31, 1.96	<0.001	1.67	1.28, 2.17	<0.001
11	1.39	1.14, 1.70	0.001	1.45	1.12, 1.87	0.004
20.1	1.50	1.26, 1.78	<0.001	1.38	1.11, 1.71	0.004
67.9	0.97	0.83, 1.14	0.70	0.97	0.80, 1.18	0.75
Length/Height						
Birth	1.14	0.97, 1.33	0.11	1.28	1.02, 1.59	0.03 c
2	1.37	1.16, 1.63	<0.001	1.38	1.11, 1.72	0.003
7	1.42	1.17, 1.72	<0.001	1.46	1.12, 1.89	0.01
11	1.42	1.16, 1.73	0.001	1.58	1.20, 2.09	0.001
20.1	1.44	1.20, 1.72	<0.001	1.61	1.25, 2.06	<0.001
67.9	1.40	1.18, 1.64	<0.001	1.54	1.22, 1.94	<0.001
Ponderal index/Body mass index						
Birth	1.17	0.99, 1.39	0.07	1.10	0.88, 1.36	0.41
2	1.31	1.10, 1.55	0.002	1.34	1.09, 1.65	0.01
7	1.34	1.11, 1.62	0.002	1.36	1.09, 1.71	0.01
11	1.18	0.97, 1.42	0.09	1.14	0.90, 1.43	0.28
20.1	1.30	1.09, 1.54	0.003	1.15	0.93, 1.41	0.19
67.9	0.84	0.71, 0.99	0.04	0.84	0.69, 1.02	0.08
Head circumference e						
Birth	1.26	1.06, 1.49	0.01	1.43	1.14, 1.79	0.002
67.9	1.26	1.07, 1.49	0.01	1.35	1.11, 1.65	0.003

Abbreviations: CI, confidence interval; OR, odds ratio.

a Logistic regression analyses were conducted as presented in Figure 1, panel A.

b Adjustments were made for length of gestation (birth measures), father's occupational status in childhood, parity, mother's age and height at delivery, history of breastfeeding, age at testing cognitive ability at 20.1 years, and time interval between tests of cognitive ability from 20.1 to 67.9 years.

c A further model adjusted for ‘b’ plus highest own achieved level of education. The marked associations were rendered non-significant. The marked associations were rendered non-significant. Unmarked associations remained as in the adjusted model ‘b’.

d A further model adjusted for ‘b’ plus diagnoses of stroke and coronary heart disease. The marked associations were rendered non-significant. Unmarked associations remained as in the adjusted model ‘b’.

e Measurements of head circumference at two, seven, 11 and 20.1 years were not available.

The odds for decline in scores from the top third relative to the odds for remaining in the top third after age 20.1 years (Figure 1, panel C) was significantly lower for men who were longer (OR = 0.76, 95% CI: 0.58 to 0.99, *P* = 0.04 for each 1 SD unit longer length; adjusted *P*-values<0.04), and had a larger head circumference at birth (the association of a larger head circumference at birth became significant after adjustments, OR = 0.69, 95% CI: 0.49 to 0.99, *P* = 0.04; unadjusted *P* = 0.21). Body size at two, seven, 11, 20,1 and 67.9 years or growth from birth to two, seven 11 and 20.1 years did not associate significantly with odds for decline in ability from the top third (*P*-values>0.07).

Men whose scores remained in the middle third did not differ in pre-or postnatal body size or in growth from men whose scores remained in the bottom third, or from men whose scores declined from the middle third to the bottom third (Figure 1, panels B and D) (*P*-values>0.07).

Finally, in a series of exploratory analyses we tested if the associations of body size at birth and at ages two, seven, 11 and 20.1 years and of growth from birth to two, seven, 11 and 20.1 with cognitive ability at 67.9 years and with cognitive decline from 20.1 years were non-linear. We found no significant non-linear effects (*P*-values>0.05).

## Discussion

Our findings showed that lower weight, length and head circumference at birth were associated with lower cognitive ability in men at a mean age of 67.9 years, and with age related decline in cognitive ability after age of 20.1 years. These associations were graded characterizing the whole range of body size at birth. Hence, our findings also showed that men who were born larger were more likely to remain in the top than in the lower two thirds in cognitive ability and were less likely to decline from than remain in the top third in cognitive ability after age 20.1 years. Our findings also revealed that slower growth in weight, height and BMI between birth and two years were associated with lower cognitive ability at 67.9 years. Men who grew faster in weight, height and BMI between birth and two years were more likely to remain in the top third in cognitive ability after age 20.1 years. However, growth between birth and two years was not associated with age related cognitive decline. These associations changed only a little when we made adjustments for length of gestation (birth size) and father's occupational status in childhood, maternal characteristics at delivery, parity, history of breastfeeding, age at and time interval between the two cognitive ability tests (change in cognitive ability). Highest own attained level of education in adulthood and diagnoses of stroke or CHD made no major changes to the findings either.

Potential limitations of our study relate to the age at which baseline cognitive status was assessed. While individuals are expected to reach their peak cognitive ability by the second or the third decade of life, some of the men in our sample, aged 17 to 28 years at the baseline cognitive assessment, may not have yet reached their individual maximum cognitive capacity. This may have attenuated, at least to some extent, the average degree of age related decline in cognitive ability up to old age observed here. We cannot rule out either that improvement as a consequence of repeating the test over several decades may still have played a role: it has been suggested that even with intervals of several years, practice may mask the other age related changes in cognitive ability [27]. On the other hand, a strength of our study is that cognitive decline could hardly be expected to have begun when baseline cognitive status in our sample was assessed. This precludes the horse racing–effect, which is often present in studies focusing on age related cognitive decline [28]. Though, a recent study suggested that cognitive decline may already be evident in middle aged populations [29]. Further, selective attrition over time may also introduce a potential bias. The current study participants were slightly larger in postnatal body size, had a higher childhood and own achieved socioeconomic background, had higher cognitive ability at mean age of 20.1 years, had survived to old age and were capable to attend the cognitive testing at 67.9 years. The Finnish Defense Forces Basic Intellectual Ability Test is designed to measure cognitive ability in young adulthood and has been used in thousands of conscripts since the 1950s. Stability of the cognitive ability across decades in our cohort was high, a finding in line with previous reports using different tests than ours [20], suggesting that there is no reason to presume that this test would not measure cognitive ability and be applicable also in older adult men. High stability, but presumably the health of the sample as well, may also have attenuated the average age related decline observed here. These restrictions, however, rather undermine than increase our ability to detect significant associations. Finally, generalizations from our study cannot be made to populations that differ in health and ethnic background and generalizations also preclude women.

Our findings agree well with previous studies that have tested associations between physical growth before and after birth with cognitive ability in children, adolescents and young adults–populations that are several decades younger than the current sample of elderly men [1]–[10]. Our findings are also in agreement with our previous report using a large sample of men of the HBCS from whom data on cognitive ability at age 20.1 years were available [11], and from whom the current sample of men were drawn to the re-test of cognitive ability nearly five decades later. The previous studies that have tested if body size at birth is associated with cognitive ability in older age have, however, produced contradictory findings. Lower cognitive ability was associated with lower birth weight in two of the previous studies [14], [15], and in one other study with lower bi-parietal head diameter, but not with weight, length, ponderal index, head circumference or occipitofrontal head diameter at birth [16]. It was not associated with weight or length at birth in one study, with head diameter in another study [17], or with head circumference or ponderal index at birth in a third study, which unfortunately did not report how cognitive ability was associated with weight or length at birth [18]. Finally, age related change in cognitive ability was not associated with weight, length and/or head circumference at birth [16], [17], [19]. Direct comparisons between the current and the existing studies in older populations are, however, complicated by differences in methodology and study design. Most importantly, none of the existing studies in the elderly have focused on postnatal growth or no previous study has had the opportunity to examine age related change in cognitive ability since young adulthood or has had the opportunity to repeat the same cognitive test battery over time.

Body size at birth and in early childhood are crude proxies of prenatal developmental milieu and early living conditions that are affected by multiple factors with potential long-term neuro-developmental consequences. The original work, supported by a number of animal experiments, has concentrated on under-nutrition as the programming insult in pregnancy [30]. Human data are scanty, but recent findings from the Dutch Hunger Winter Study have indeed demonstrated that severe under-nutrition during early stages of gestation was associated with a lower score on a test measuring selective attention in the offspring in mid-life [31]. Our study group has recently demonstrated that hypertension spectrum disorders during pregnancy, one of the key determinants of slow intrauterine growth, is associated with poorer cognitive function and greater cognitive decline in a subsample of the men participating in the current study [32], [33]. Substantial evidence from animals studies support the role of fetal overexposure to glucocorticoids as another programming insult in pregnancy [34]. As the maternal and fetal cortisol levels are highly correlated it is not surprising that higher maternal cortisol during pregnancy and higher maternal prenatal stress as its surrogate marker, have been related to poorer cognitive function in the offspring in childhood [35]. One study has even demonstrated that higher maternal consumption of glycyrrhizin in licorice during pregnancy, an inhibitor of the placental glucocorticoid barrier mechanism, is associated with poorer cognitive function in the offspring in childhood [36]. Other underlying mechanisms may relate to infections, genetic and epigenetic mechanisms [37], [38], and also suboptimal parenting. While our study did control for father's occupational status in childhood, a possibility remains that lower socioeconomic background may still operate behind the associations. Indeed, slower growth particularly in height is an accepted indicator of childhood socioeconomic adversities [39]. It is however worth noting that growth during infancy may be a more sensitive marker of childhood living conditions than childhood growth. An infant invests up to 40% of its energy for growth [39]. This falls to around two percent after two years [40]. Infants more readily divert energy away from growth to meet other needs, such as to combat infection, than do older children. The lesser effect of growth after the age of two years on cognitive ability could therefore reflect its lesser sensitivity as a marker of living conditions, rather than the lesser importance of this phase of life for cognitive development. Further research efforts are needed that concentrate on finding the biological mechanisms that potentially underlie the associations reported here.

In light of clinical importance of the findings, the associations detected by our study could be considered relatively modest. Studying early determinants and predictors of cognitive aging has, however, been repeatedly emphasized [29] and identified as a research priority [41]. Thus, even if relatively modest in effect size, our findings contribute significantly to this research and suggest that age related change in cognitive decline may have origins in early pre-and postnatal life.
